# Prebiotics and Gut Health: Mechanisms, Clinical Evidence, and Future Directions

**DOI:** 10.3390/nu18030372

**Published:** 2026-01-23

**Authors:** Cinara Regina A. V. Monteiro, Eduarda G. Bogea, Carmem D. L. Campos, José L. Pereira-Filho, Viviane S. S. Almeida, André A. M. Vale, Ana Paula S. Azevedo-Santos, Valério Monteiro-Neto

**Affiliations:** 1 Center of Biological and Health Sciences, Federal University of Maranhão, Bacanga Campus, São Luís 65080-805, MA, Brazil; cinara.monteiro@discente.ufma.br (C.R.A.V.M.); eduardabogea@gmail.com (E.G.B.); carmem.campos@discente.ufma.br (C.D.L.C.); jlp.filho@discente.ufma.br (J.L.P.-F.); viviane.almeida@discente.ufma.br (V.S.S.A.); andre_amvale@hotmail.com (A.A.M.V.); ana.azevedo@ufma.br (A.P.S.A.-S.); 2 School of Physical Therapy, Florence University Center, São Luís 65.020-490, MA, Brazil

**Keywords:** prebiotics, gut microbiota, short-chain fatty acids (SCFAs), intestinal barrier, randomized controlled trials, personalized nutrition, synbiotics, gastrointestinal health

## Abstract

Background/Objectives: Prebiotics, which are non-digestible compounds that selectively modulate gut microbiota, are recognized for their potential to promote host health. Although their bifidogenic effect is well documented, a systematic synthesis of how this microbial modulation translates into clinical gastrointestinal (GI) and metabolic outcomes across diverse populations is needed. This review aims to integrate mechanistic insights with clinical evidence to elucidate the pathway from prebiotic structures to tangible health benefits. Methods: This comprehensive narrative review details the structural properties of major prebiotics (e.g., inulin, FOS, and GOS) that govern their fermentation and the production of short-chain fatty acids (SCFAs). To evaluate clinical efficacy, an analysis of 22 randomized controlled trials from the past decade was conducted, focusing on human studies that utilized ISAPP-recognized prebiotics as the sole intervention. Results: The analysis confirms that prebiotic supplementation consistently increased the abundance of beneficial bacteria (e.g., *Bifidobacterium* and *Lactobacillus*) and SCFA production. These changes are associated with significant clinical improvements, including enhanced stool frequency and consistency, strengthened intestinal barrier function, and modulated immune responses. Benefits have been documented in healthy individuals, children, the elderly, and those with conditions such as constipation, metabolic syndrome, and antibiotic-associated dysbiosis. However, significant inter-individual variability in response was evident, and the study designs showed notable heterogeneity in prebiotic type, dosage, and duration. Conclusions: Prebiotics are effective modulators of gut health, driving clinical benefits through selective microbial fermentation and SCFA production. The documented heterogeneity and variability highlight the need for future research to focus on personalized nutritional strategies. Key priorities include standardizing intervention protocols, elucidating dose–response relationships, integrating multi-omics data to link taxonomy to function, and exploring novel applications such as synbiotic formulations and gut–brain axis modulation.

## 1. Introduction

Gut health is a critical factor in overall well-being and plays a pivotal role in maintaining optimal physiological function [1,2,3]. The complex relationships among diet, gut microbiota, and health have garnered significant attention in recent years. Research has shown that the composition and activity of the gut microbiota can influence various physiological processes, including immune function, metabolism, and mental health [2,3,4,5]. Furthermore, an imbalance in the gut microbiota, known as dysbiosis, has been linked to the development of a range of disorders and diseases, such as inflammatory bowel disease, obesity, and neuropsychiatric disorders [6,7,8]. This has led to a growing interest in developing strategies to promote a healthy gut microbiota and restore balance when dysbiosis occurs.

In recent years, there has been a surge in research exploring the potential of prebiotics to support and enhance gut health [1,9,10,11]. This, in turn, can lead to a more diverse and resilient gut microbiota, which is associated with positive health outcomes. Understanding the intricate interplay between prebiotics, gut microbiota, and human health holds great promise for developing targeted interventions to improve gut health and overall well-being. Furthermore, unraveling the mechanisms through which prebiotics exert their effects on the gut microbiota can provide valuable insights into potential therapeutic strategies for combating dysbiosis-related conditions [1].

Although the bifidogenic effect of prebiotics is well documented, a critical synthesis that systematically links this microbial modulation to concrete clinical gastrointestinal and metabolic outcomes across diverse human populations remains unexplored. Current literature often lacks an integrative analysis that bridges mechanistic insights rooted in prebiotic structure and function with heterogeneous clinical evidence derived from human trials. Therefore, this review aims to address this gap by critically synthesizing findings from randomized controlled trials published over the last decade, with a specific focus on elucidating the translational pathway from selective microbial modulation to tangible health benefits in the host. Furthermore, we provide a detailed examination of prebiotic structural properties, discuss the determinants of inter-individual response variability, and propose a framework for advancing personalized prebiotic interventions in the future.

## 2. Methodology

This narrative review synthesizes the current knowledge on prebiotics, with a critical focus on their mechanisms and clinical applications. To ensure a robust analysis of clinical efficacy, a systematic search strategy was employed to identify relevant randomized controlled trials (RCTs) and other interventional clinical studies in humans. The findings of this analysis are presented and synthesized in Section 6.

For the selection of studies included in this section, literature searches were conducted in Scopus and PubMed using the following keywords alone or in combination: prebiotics, gastrointestinal disorders, metabolic disorders, prebiotic effects, and gut microbiota.

The inclusion criteria were as follows:

i. Full-text articles published in English within the last 10 years.
ii. Human clinical trials, including randomized controlled trials (RCTs), phase I–IV trials, and multicenter studies.
iii. Studies evaluating the impact of prebiotics recognized by the International Scientific Association for Probiotics and Prebiotics (ISAPP) as a sole intervention on intestinal or metabolic health outcomes.

The exclusion criteria were as follows:

i. Trials using probiotics, synbiotics, or postbiotics.
ii. Trials employing prebiotic supplements that are not recognized by the ISAPP consensus.
iii. Studies that used only foods with prebiotic potential without specifying the prebiotic compounds.
iv. Studies in which a prebiotic was used as a placebo comparator.

## 3. Definition of Prebiotics

The term prebiotic was first proposed to describe non-digestible food ingredients that beneficially affect the host by selectively stimulating the growth and/or activity of specific bacteria in the colon. With advances in microbiome research, this concept has been broadened to include substrates that act throughout the gastrointestinal tract [12,13]. According to the International Scientific Association for Probiotics and Prebiotics (ISAPP), a prebiotic is defined as “a substrate that is selectively utilized by host microorganisms conferring a health benefit.” This definition highlights two key characteristics: selective utilization by beneficial microorganisms and evidence of physiological benefits to the host [13].

Naturally occurring prebiotics include compounds found in fruits, vegetables, and whole grains, whereas purified forms, such as inulin, fructo-oligosaccharides (FOSs), and galacto-oligosaccharides (GOSs), are used in nutritional products. These carbohydrates resist digestion in the upper gastrointestinal tract and are fermented by specific microbial groups, generating short-chain fatty acids and other metabolites involved in intestinal and metabolic regulation [12,14].

Prebiotics differ from probiotics, which are live microorganisms that confer health benefits when consumed in adequate amounts. In contrast, prebiotics are non-living substrates that enhance the growth or activity of beneficial bacteria [15]. The inclusion of these compounds in foods and dietary supplements is consistent with their biological role as modulators of gut microbial composition and function. Prebiotics can be used in various applications, including food and beverages, dietary supplements, and animal feed [12,16,17]. They offer a way to enhance the nutritional value of products and promote overall health without compromising their taste or quality. Beyond their physiological definition, the practical application of prebiotics in functional foods, dietary supplements, and clinical nutrition requires careful consideration of the following factors: specific prebiotic compounds, dosage, stability during processing and storage, and compatibility with the food or supplement matrix. These considerations are crucial for ensuring that prebiotic-containing products deliver the intended selective modulation of the gut microbiota in vivo.

## 4. Classification and Structural Properties of Prebiotics

Prebiotics can be classified into different categories based on their chemical structure and sources. These classifications provide insights into their diverse functions and possible applications in terms of gut health and nutrition. To classify prebiotics based on their chemical structure, it is crucial to examine the specific mechanisms by which they interact with gut microbiota [12].

Moreover, classifying prebiotics based on their origin offers a wide array of opportunities to explore the potential health benefits of plant-derived prebiotics. Investigating the presence of prebiotic fibers, such as inulin, fructo-oligosaccharides, and resistant starch, in various fruits, vegetables, and roots enables a deeper understanding of their natural sources and potential applications [14,18]. Examples of prebiotics within each category include the following:

### 4.1. Classification by Chemical Structure

**Figure 1:**
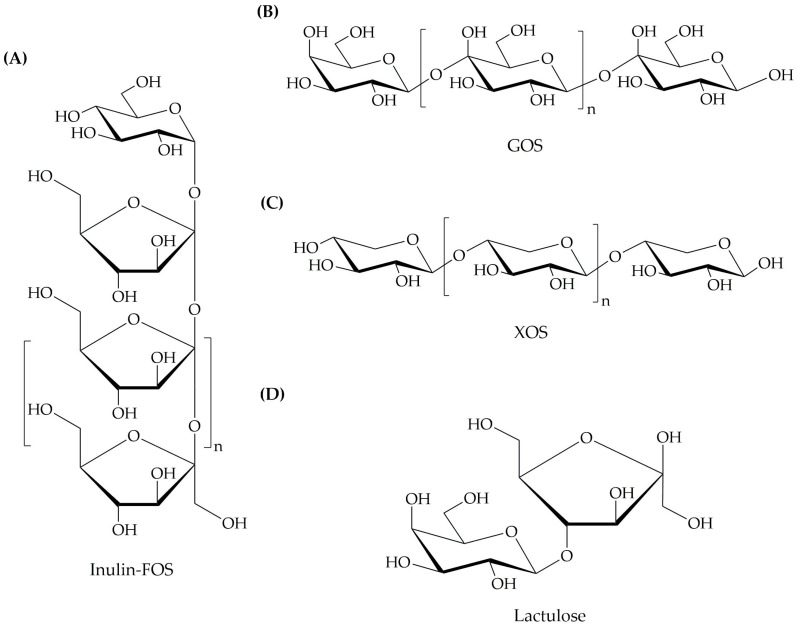
Chemical structures and linkage configurations of representative prebiotic carbohydrates. The selective fermentation of these compounds is determined by their specific glycosidic bonds, which provide resistance to human digestive enzymes: (**A**) Inulin and Fructo-oligosaccharides (FOSs): linear chains of fructose units joined by β(2→1) glycosidic linkages, where “n” represents the variable number of repeating units (degree of polymerization, or DP ~2–60 for inulin and DP ~2–10 for FOS). (**B**) Galacto-oligosaccharides (GOSs), galactose-based chains featuring β(1→4) and β(1→6) bonds. (**C**) Xylo-oligosaccharides (XOSs) are characterized by xylose units linked by β(1→4) glycosidic bonds. (**D**) Lactulose is a synthetic disaccharide composed of galactose and fructose joined by a β (1→4) linkage. These structural configurations ensure that the molecules reach the colon intact for utilization by beneficial microbes. Source: The chemical structures were rendered by the authors using ChemDraw 18.1 (PerkinElmer Informatics, Waltham, MA, USA), adapted from previously published chemical structures [19,20,21,22,23].

The chemical structure of a prebiotic fundamentally dictates its physical properties, fermentability, and interactions with the gut microbiota. Prebiotics are primarily categorized into oligosaccharides, polysaccharides, and non-carbohydrate compounds based on their molecular architecture [13]. The distinct molecular architectures of these prebiotic classes directly influence their physical properties, fermentability, and selective utilization by gut microbes. The representative chemical structures of the key prebiotic compounds are illustrated in Figure 1.

#### 4.1.1. Oligosaccharides

Oligosaccharides are short-chain carbohydrates that typically comprise 3–10 monosaccharide units linked by glycosidic bonds [17]. Their relatively small size and specific bond configurations render them largely resistant to hydrolysis by human digestive enzymes, allowing them to reach the colon intact. This category includes some of the most well-researched prebiotics:

- Fructo-oligosaccharides (FOSs) and inulin-type fructans (ITFs): Characterized by β(2→1) linkages between fructose units. They exhibit high solubility in water and are rapidly fermented, primarily by bifidobacteria.
- Galacto-oligosaccharides (GOSs): Composed of galactose units linked by β(1→4) or β(1→6) bonds, often with a terminal glucose unit. GOSs are notably bifidogenic and are found naturally in human milk (as human milk oligosaccharides, HMOs).

Their selective fermentability by beneficial bacteria makes oligosaccharides a cornerstone of classic prebiotic supplementation.

#### 4.1.2. Polysaccharides

Polysaccharide prebiotics are larger and more complex carbohydrates consisting of long chains (often hundreds) of monosaccharide units. Their higher molecular weight and often branched or crystalline structures contribute to slower and more variable fermentation kinetics in the colon [17,24,25].

- Resistant starch (RS): Encompasses several types (RS1–RS4) of starch that escape digestion. Its fermentation promotes the growth of bacteria, such as *Ruminococcus* and *Eubacterium*, leading to significant butyrate production.
- Arabinoxylan: A hemicellulose found in cereal grains, consisting of a xylose backbone with arabinose side chains. Its complex structure supports a broader range of microbial degraders and fosters the production of various SCFAs.

Gradual fermentation of polysaccharides provides a sustained supply of metabolic substrates to the distal colon, supporting microbial diversity and stability.

#### 4.1.3. Non-Carbohydrate Prebiotics

Beyond carbohydrates, certain other dietary compounds demonstrate prebiotic activity by selectively modulating the gut microbiota, although they are often termed “prebiotic candidates” pending further clinical validation [18,26].

- Polyphenols: Abundant in plant foods such as green tea, berries, and cocoa, these compounds (e.g., flavonoids and phenolic acids) are poorly absorbed. They can inhibit pathogenic bacteria while stimulating the growth of beneficial *Lactobacillus* and *Bifidobacterium*, partly through microbial biotransformation.
- Amino Acids and Derivatives: Specific amino acids, such as glutamine and arginine, as well as conjugated linoleic acid (CLA), may serve as selective substrates or signaling molecules for certain gut microbes, influencing the microbial community structure and host immune function.

### 4.2. Classification by Origin

#### 4.2.1. Plant-Derived Prebiotics

Many prebiotics are naturally present in plants, including chicory root, Jerusalem artichoke, and various fruits and vegetables. These plants contain high amounts of prebiotic fibers, such as inulin, FOS, and resistant starch [18,26,27].

#### 4.2.2. Animal-Derived Prebiotics

Some oligosaccharides are derived from animal sources and have been found to exert beneficial effects on gut health. Research in this area has also highlighted the potential benefits of animal-derived prebiotics, particularly milk oligosaccharides, in promoting gut health. Furthermore, the exploration of novel prebiotic sources from marine sources, fungi, and other natural sources presents an exciting opportunity to expand the range of available prebiotic compounds [18,28,29,30,31].

Other compounds with prebiotic potential include pectin, β-glucans, and xylo-oligosaccharides [12,32,33]. Natural sources of prebiotics are abundant, and various foods and plants contain these beneficial compounds, including garlic, onion, barley, artichoke, chicory, dandelion greens, chia seeds, almonds, oats, and flaxseeds [12,18].

### 4.3. Structure–Function Relationships

Prebiotics are characterized by their resistance to enzymatic breakdown in the upper gastrointestinal tract, allowing them to reach the colon [34,35]. By examining the molecular conformation, glycosidic linkages, and branching patterns of prebiotics, we can uncover how these structural features influence their interactions with gut microbiota [12,18].

The resistance of prebiotics to digestion is attributed to their unique chemical structures, particularly the nature of the glycosidic linkages connecting sugar molecules. Enzymes present in the upper gastrointestinal tract, such as amylase and sucrase, break down carbohydrates into smaller, absorbable sugars [34]. However, β-glycosidic linkages found in prebiotics are less susceptible to enzymatic cleavage than the α-glycosidic linkages typically found in digestible carbohydrates [34]. Research has shown that the specific arrangement of monosaccharides in prebiotics can affect their fermentation properties and ability to selectively stimulate the growth of beneficial bacteria [36,37,38].

Inulin, a commonly studied prebiotic, is a linear fructose polymer with β(2→1) glycosidic linkages. The presence of β(2→1) linkages endows inulin with unique structural properties, such as resistance to digestion by human enzymes but susceptibility to selective fermentation by specific saccharolytic gut bacteria, such as *Bifidobacterium*, *Lactobacillus*, *Anaerostipes*, and *Faecalibacterium* [39,40]. In contrast, fructo-oligosaccharides consist of a chain of fructose units linked by β(2→1) glycosidic bonds, similar to inulin. However, FOS has a lower degree of polymerization and shorter chain length than inulin [39,41]. GOS is another type of prebiotic with a more complex structure. They are composed of chains of galactose units linked by β(1→4) or β(1→6) glycosidic linkages. These glycosidic linkages confer different properties to GOS and influence their fermentation by gut bacteria [42].

Furthermore, the branching patterns of prebiotics can influence their behavior. For example, xylo-oligosaccharides (XOSs) contain a backbone of xylose units with branching patterns created by β-1,4-linked xylose. These branching patterns can affect the solubility, stability, and fermentability of XOSs [43]. Overall, the structural features of prebiotics play crucial roles in determining their functionality and behavior [44,45,46,47].

## 5. Beneficial Effects of Prebiotics

As described above, owing to their structural characteristics, prebiotics pass through the upper gastrointestinal tract relatively intact, reaching the large intestine, where they can serve as selective substrates for the growth and proliferation of beneficial gut microorganisms, particularly *Bifidobacterium* and *Lactobacillus* species [34,35]. These shifts in gut microbiota composition are associated with various health benefits, including competition with pathogens for adhesion to the gut epithelium, production of short-chain fatty acids (SCFAs), and immune function modulation [1,48].

These microorganisms possess metabolic machinery that enables them to utilize prebiotics as carbon and energy sources through a process known as fermentation, resulting in the production of SCFAs such as acetate, propionate, and butyrate [49,50,51,52]. Notably, the major fermentation end products of *Bifidobacterium* and *Lactobacillus* when grown in pure culture are lactate and acetate, which are subsequently utilized by butyrogenic bacteria, such as *Eubacterium*, *Faecalibacterium*, and *Roseburia*, to produce butyrate in a process called cross-feeding [48,53,54].

Among the SCFAs, butyrate plays a crucial role in providing energy to intestinal cells, as it is absorbed and utilized as fuel by colonic cells. Propionate can be converted into glucose via intestinal gluconeogenesis [55] or diffused into the portal vein for hepatic gluconeogenesis [56]. More than 90% of SCFAs are absorbed by the gut and utilized by the microbiota. However, only small amounts of SCFAs, primarily propionate and acetate, were detected in the peripheral circulation. Acetate is the most abundant SCFA found in circulation and has been shown to cross the blood–brain barrier (BBB) [57].

In addition, SCFAs exert numerous physiological effects on the host. They contribute to maintaining gut health by improving the integrity of the colonic epithelium and promoting the growth and differentiation of colonic cells. Furthermore, SCFAs have anti-inflammatory properties and can modulate the immune response, thereby affecting various physiological processes [49,52,58,59,60,61].

Notably, the fermentation of prebiotics in the human gut not only influences the composition and metabolic activity of the microbial community but also contributes to the overall gut microbiota balance. SCFA production is linked to a reduction in the pH of the colonic environment, creating an environment that is less favorable for the growth of potentially pathogenic microorganisms. This, in turn, promotes the dominance of beneficial gut bacteria [62,63]. A decrease in pH can increase the absorption of calcium from the intestines, which is influenced by factors such as the solubility of calcium and pH in different parts of the gastrointestinal tract [64]. Furthermore, SCFAs may stimulate the expression of intracellular calcium transporter genes [65]. The bioavailability of other minerals, such as iron and magnesium, is also enhanced by the prebiotic consumption [66,67].

**Figure 2:**
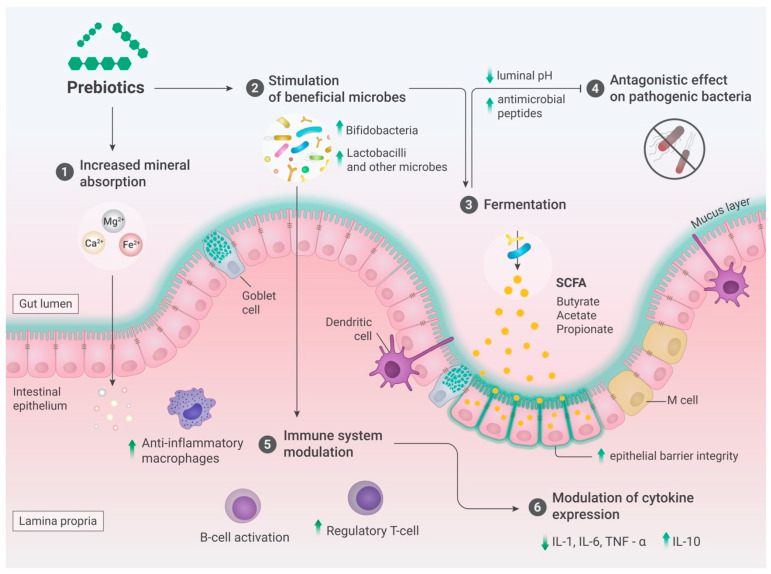
Mechanisms of prebiotic action. The health effects of prebiotics are multifaceted and involve several mechanisms. These mechanisms include (1) optimization of the absorption of minerals such as calcium, iron, and magnesium, which are transferred from the small intestine to the large intestine and osmotically affected, making them more soluble; (2) promotion of the growth of beneficial microbes by providing a suitable substrate; (3) enhanced production of metabolic substances, such as SCFAs, which are utilized as energy substrates for colonocytes, contribute to the production of anti-inflammatory mediators, and support the integrity of the intestinal mucosa; (4) inhibition of athogen growth and/or elimination by reducing luminal pH (through the production of acetic and lactic acids) and generating antimicrobial peptides; (5) modulation of the immune system through the maintenance of the epithelial barrier’s integrity and the modulation of innate immunity, involving changes in macrophage polarization and function, activation of B cells and subsequent antibody release, increased dendritic cell activity, and regulatory T-cell differentiation; and (6) modulation of various cytokines, including inhibition of pro-inflammatory marker secretion (IL-1, IL-6, and TNF-alpha) and increased expression of anti-inflammatory cytokines, such as IL-10. ↑: Increase, ↓: Decrease. Source: The figure was custom-created by a professional scientific illustrator (www.scivisual.com).

Moreover, the impact of SCFAs extends beyond the gut, as they have been shown to interact with various organs and systems in the body [48,51,68,69]. For instance, SCFAs can affect lipid metabolism, glucose homeostasis, and appetite regulation, linking the gut microbiota and prebiotic fermentation to metabolic health and potentially influencing conditions such as obesity and type 2 diabetes [1,6,58,69,70,71]. Prebiotics play a crucial role in maintaining gut health and fostering a balanced microbial ecosystem, thereby influencing various physiological processes. In addition to SCFAs, many probiotic strains produce antimicrobial peptides and other compounds, such as bacteriocins and H_2_O_2_, which exert antagonistic effects on pathogenic bacteria [72]. A summary of these multifaceted mechanisms is presented in Figure 2.

Emerging evidence underscores that the benefits of prebiotics extend beyond gastrointestinal and metabolic health to influence brain function and mental well-being through the gut–brain axis. The modulation of gut microbiota composition and the subsequent increase in SCFA production (particularly butyrate and acetate) by prebiotic fermentation are central to this communication. SCFAs can cross the BBB, exert neuroactive properties, reduce neuroinflammation, and influence the expression of brain-derived neurotrophic factor (BDNF) [7,73]. Furthermore, prebiotic-induced changes in gut microbiota can regulate the production of neurotransmitters (e.g., serotonin and gamma-aminobutyric acid—GABA) and modulate the activity of the hypothalamic–pituitary–adrenal (HPA) axis, thereby impacting stress responses, mood, and cognitive function [74,75]. Mechanistic insights from animal models have further clarified these pathways. For instance, in mice fed a high-fat diet (HFD), the administration of a FOS and GOS mixture ameliorated anxiety- and depression-like behaviors by restoring intestinal barrier integrity and reducing pro-inflammatory cytokines, such as TNF-α and IL-1β, in the hippocampus [76]. While promising, clinical evidence in humans is still evolving, and future research is needed to fully elucidate specific prebiotic pathways and their therapeutic potential for neuropsychiatric and neurodegenerative conditions.

As we delve deeper into the intricate mechanisms of prebiotic digestion and fermentation in the human gut, it becomes evident that the effects of prebiotics extend far beyond the gastrointestinal tract. Understanding the holistic impact of prebiotics and their fermentation products on the human body offers valuable insights into the potential therapeutic use of prebiotics in the prevention of various diseases.

## 6. Effects of Prebiotics on the Human Gastrointestinal Tract: From Microbial Shifts to Clinical Outcomes

### 6.1. Modulation of Gut Microbiota Composition and Inter-Individual Variability

The most fundamental and consistently documented action of prebiotics is the selective modulation of the gut microbiota, particularly the promotion of symbiotic bacteria such as *Bifidobacterium* and *Lactobacillus*. However, this bifidogenic effect is not uniform and is significantly influenced by an individual’s baseline microbial ecosystem. Johnson et al. demonstrated that the response to prebiotic consumption can vary depending on the baseline composition of an individual’s gut microbiota [77]. Understanding these inter-individual differences is crucial for personalized nutrition and targeted interventions to promote healthy gut microbiota.

Overall, the literature underscores the intricate relationship between prebiotic consumption and gut microbiota composition, emphasizing the need for further research to elucidate specific mechanisms and individualized responses. These studies suggest that prebiotic consumption can lead to shifts in gut microbiota composition, characterized by an increase in beneficial bacteria and a potential decrease in the abundance of pathogenic bacteria. Diverse and balanced gut microbiota are essential for overall health [13].

Despite the lack of broad-scale compositional changes, supplementation with prebiotics, such as FOS, inulin, XOS, and GOS, has increased the abundance of health-promoting lactic acid bacteria, particularly *Bifidobacterium* and *Lactobacillus* [24,60,78,79]. While the literature underscores the potential benefits of prebiotics in modifying the gut microbiota composition, it is essential to recognize the limitations inherent in these studies. Some data suggest that the effects of prebiotics on the gut microbiota are more complex than initially assumed. The response to prebiotic supplementation is notably variable among individuals, a phenomenon largely attributed to differences in baseline gut microbiota composition [77,78].

Although some individuals experienced an increase in beneficial bacteria, others showed no significant changes or even a decrease in the number of these populations [80]. This variability raises questions regarding the reliability of prebiotics as a strategy to promote the growth of beneficial gut bacteria. For instance, in ulcerative colitis (UC), the bifidogenic effect of GOS was observed only in patients with less active disease at baseline, highlighting how the inflammatory milieu can preclude a prebiotic response [81]. Additionally, the long-term effects of sustained prebiotic consumption on gut microbiota composition remain unclear [82]. Some studies have indicated that prolonged exposure to certain prebiotics may lead to potential imbalances in the gut microbiota, including the overgrowth of specific bacterial species, which could have adverse effects on gut health. Furthermore, the effectiveness and safety of prebiotics compared to probiotics are still under investigation [12,42].

Furthermore, the potential impact of prebiotics on the gut microbiota of individuals with pre-existing conditions such as inflammatory bowel disease (IBD) or irritable bowel syndrome (IBS) must be carefully considered. Evidence suggests that prebiotic consumption may not yield the same benefits in individuals with underlying gut health issues and may exacerbate their symptoms [3,15].

In light of these conflicting findings, more comprehensive and rigorous research is necessary to fully understand the effects of prebiotic consumption on the gut microbiota composition. Future studies should address individual variations in response to prebiotics, potential long-term effects, and the influence of prebiotics on individuals with different health conditions. Without overlooking these opposing perspectives, we can gain a more balanced and nuanced understanding of the relationship between prebiotics and gut microbiota composition.

The most fundamental and consistently documented action of prebiotics is the selective modulation of the gut microbiota, particularly the promotion of symbiotic bacteria such as *Bifidobacterium* and *Lactobacillus*. However, this bifidogenic effect is not uniform and is significantly influenced by an individual’s baseline microbial ecosystem and other host-specific factors. The considerable inter-individual variability observed in response to prebiotic interventions is a critical determinant of their clinical efficacy and stems from several key factors.

The most prominent driver is the baseline composition and functional capacity of the gut microbiota. An individual’s microbial community dictates the available enzymatic machinery (e.g., β-fructosidases and β-galactosidases) required to initially hydrolyze specific prebiotic structures. Therefore, the presence and abundance of primary degraders (such as certain *Bifidobacterium* species) at baseline can predict the magnitude of the subsequent response. As demonstrated by Johnson et al., the response to dietary interventions, including prebiotics, is highly dependent on the initial microbial configuration [77]. This principle is mechanistically supported by studies showing that the in vitro fermentability of prebiotics, such as inulin and FOS, directly correlates with the baseline levels of bifidobacteria in fecal inocula [83,84]. Furthermore, the concept of “microbial enterotypes” or community state types may influence ecological outcomes; for instance, individuals with a *Prevotella*-rich enterotype might metabolize fiber and prebiotics differently than those with a *Bacteroides*-rich community [85].

The host’s physiological and pathological states are closely linked to microbiota composition. Systemic and local inflammation can profoundly alter the gut environment and microbial metabolism, thereby dampening prebiotic effects. This is exemplified in UC, where the bifidogenic effect of GOS was observed only in patients with less active disease at baseline, highlighting how the inflammatory milieu can preclude a prebiotic response [81]. Similarly, conditions such as obesity and type 2 diabetes, which are characterized by low-grade inflammation and distinct dysbiosis, may require different prebiotic strategies or doses to elicit a meaningful shift [86].

Genetic and epigenetic host factors also play foundational roles. Host genotypes can influence mucosal glycosylation patterns, secretion of antimicrobial peptides, and innate immune responses, all of which shape the habitat available to both resident and incoming microbes. Although direct human studies linking specific genetic polymorphisms to prebiotic responses are still emerging, animal models suggest that host genetics are a major determinant of microbiota composition [87]. Additionally, long-term dietary patterns serve as powerful modulators of the baseline microbiota and its metabolic potential. Habitual high intake of dietary fiber may lead to an enrichment of saccharolytic bacteria, potentially creating a “saturated” ecosystem where the added effect of a prebiotic supplement is marginal compared to its impact in a low-fiber, dysbiotic state [88]. This underscores the importance of considering the background diet as a key variable.

Finally, prebiotic-specific factors, including chemical structure, dose, and duration of intake, interact with these host variables. A dose that is effective in one individual may be suboptimal or excessive in another, depending on the microbial metabolic capacity. The phenomenon of “non-responders” in prebiotic trials may not indicate inefficacy but rather a mismatch between the prebiotic compound, its dosage, and the individual’s unique microbial and physiological context [89].

In conclusion, the response to prebiotics is governed by a complex interplay between (i) the initial taxonomic and functional landscape of the gut microbiota, (ii) the host’s health status and inflammatory tone, (iii) underlying host genetics and habitual diet, and (iv) the specific properties of the prebiotic intervention. This variability is not merely noise but a central feature that must be addressed to move from generic recommendations to personalized nutritional strategies. Future research must integrate multi-omics approaches (metagenomics and metabolomics) with detailed host phenotyping to identify predictive biomarkers of response, enabling the targeted application of specific prebiotics to those most likely to benefit.

### 6.2. Clinical Evidence: Analysis of Randomized Controlled Trials and Other Clinical Studies

To assess the clinical effects of prebiotics on gastrointestinal and metabolic health, a systematic search of clinical studies was performed. The initial search yielded 879 records, of which 22 randomized controlled trials and interventional studies met the inclusion and exclusion criteria and were retained for in-depth analyses. The characteristics and primary outcomes of these studies are summarized in Table 1. This evidence provides a comprehensive and nuanced understanding of how prebiotics directly modulate the human gastrointestinal (GI) tract. The collected data delineate a coherent pathway from the selective reshaping of the gut microbiota and production of key functional metabolites to tangible improvements in intestinal barrier function, mucosal immunity, and the management of specific clinical GI conditions. Therefore, this analysis moves beyond merely cataloging the bifidogenic effect to explain how this ecological shift translates into concrete benefits for gastrointestinal health.

### 6.3. Key Gastrointestinal and Metabolic Outcomes

**Figure 3:**
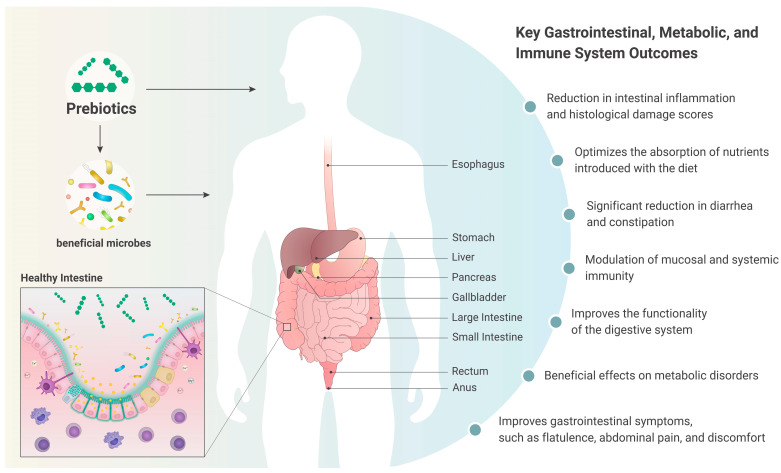
Key gastrointestinal, metabolic, and immunological outcomes of prebiotic intake. Prebiotic supplementation modulates gut microbiota and SCFA production, leading to integrated benefits across the digestive tract (e.g., improved motility and reduced inflammation), metabolic health, and systemic immunity, as evidenced by the clinical trials summarized in this review. Source: The figure was custom-created by a professional scientific illustrator (www.scivisual.com).

Selective modulation of the gut microbiota by prebiotics initiates a cascade of local and systemic effects. The clinical evidence synthesized in Table 1 demonstrates that these microbial changes translate into measurable improvements in several core domains of gastrointestinal and metabolic health. The selective modulation of the gut microbiota by prebiotics initiates a cascade of local and systemic effects, resulting in measurable improvements across the gastrointestinal, metabolic, and immune domains, as summarized in Figure 3.

#### 6.3.1. Improvement in Core Intestinal Functions: Motility and Barrier Integrity

A direct consequence of prebiotic fermentation is the enhancement of core intestinal physiology, notably through improved colonic transit and reinforcement of intestinal epithelial barriers. These effects are largely mediated by microbial metabolites, especially SCFAs (Figure 2), resulting from the selective promotion of symbiotic bacteria, most prominently *Bifidobacterium*. This bifidogenic effect has been demonstrated in diverse populations, including healthy adults [107], the elderly [105,106], children [90,91,93], and individuals with other disorders such as type 2 diabetes [101].

This modulation extends beyond a single genus. For instance, an increase in the butyrate-producing *Anaerostipes* has been reported [78,99]. Notably, Birkeland et al. [101] reported a significant bifidogenic effect, particularly an increase in *Bifidobacterium adolescentis*, along with elevated fecal SCFA levels in patients with type 2 diabetes, whereas Biruete et al. [102] observed an increase in *Akkermansia* following inulin supplementation in hemodialysis patients. Neither study reported a significant increase in *Faecalibacterium prausnitzii*. This shift toward a saccharolytic, SCFA-producing consortium is often accompanied by the suppression of less desirable taxa, such as the bile acid-metabolizing, potentially pro-inflammatory genus *Bilophila*, which has been linked to clinical improvements [78].

Prebiotics have the potential for ecological stabilization in dysbiosis. They effectively attenuate antibiotic-induced collapse in *Bifidobacterium* and blooms of Enterobacteriaceae, as shown in children by Soldi et al. [93], and promote rapid microbial recovery after antibiotic treatment [108].

This directed microbial alteration drives the most immediate functional outcome: enhancement of core digestive and intestinal function. The physical and osmotic effects of fermentation directly improved colonic transit, as evidenced by increased stool frequency and/or softer consistency in the diverse groups. These groups include healthy low-fiber consumers [99], adults with self-reported constipation [78,98], infants with constipation [94], and children with celiac disease on a gluten-free diet [90]. Notably, prebiotic supplementation also normalizes stool form and reduces stool urgency in adults with active UC, indicating a symptom-modifying effect, even in the context of inflammation [81]. This principle also applies to critical care settings beyond chronic management. For instance, in moderate-to-severe acute pancreatitis, Wang et al. [11] showed that lactulose administration significantly improved intestinal dysfunction scores in moderate-to-severe acute pancreatitis, which is a critical step in patient management. Similarly, in pediatric respiratory infections, the co-administration of lactulose with azithromycin promoted faster microbiome recovery, demonstrating its stabilizing role during antibiotic therapy [108].

A paramount gastrointestinal benefit of prebiotic use is the reinforcement of intestinal barrier integrity, largely mediated by SCFAs, particularly butyrate. Biruete et al. [102] provided direct evidence for this mechanism in hemodialysis patients, where inulin supplementation increased fecal butyrate concentration, a metabolic output known to support colonocyte health and tight-junction integrity. Functional proof of improved barrier function comes from Ho et al. [92], who reported that GOS supplementation in children with type 1 diabetes (T1D) led to a significant reduction in intestinal permeability. Additionally, the prebiotic-induced microbial shift away from pro-inflammatory taxa, such as *Bilophila* [78], further contributes to a luminal environment that supports epithelial integrity.

In addition to improving intestinal function, prebiotics directly engage with the host immune system at the mucosal interface.

#### 6.3.2. Modulation of Mucosal and Systemic Immunity

In addition to their role in digestive function, prebiotics exert direct immunomodulatory effects on the gut mucosa. Prebiotics exert direct immunomodulatory effects on the gut mucosa. Acting as dietary ligands for gut-associated lymphoid tissues, they prime both innate and adaptive immune responses, leading to enhanced frontline defense. Clarke et al. [104] demonstrated that β(2-1) fructan increases fecal secretory IgA (sIgA), a key first-line antibody defense, and enhances the expression of pathogen-sensing receptors (TLR2/4) on immune cells. Similarly, Vulevic et al. [105] observed that GOS boosted natural killer (NK) cell activity in elderly participants. This local immunomodulatory effect translates into tangible clinical benefits, such as a reduced incidence of febrile episodes and antibiotic needs in children during the cold season, as observed by Soldi et al. [93].

The combined improvement in barrier function and immune modulation provides a foundation for the application of prebiotics in specific disease contexts, such as IBD.

#### 6.3.3. Management of Specific Clinical Conditions

The convergence of improved gut motility, barrier function, and immunomodulation provides a rational basis for the application of prebiotics in specific clinical settings:

- Antibiotic-Associated and Pediatric Dysbiosis: Prebiotics act as ecological buffers, preserving commensal communities. In children, these compounds attenuate antibiotic-induced declines in *Bifidobacterium* and reduce the incidence of infection [93]. Furthermore, lactulose co-administered with azithromycin promoted a more favorable and faster microbial recovery than antibiotics alone [108].
- Acute Gastrointestinal Injury: Prebiotics may play a protective role by maintaining a beneficial SCFA-producing microbiota. In patients undergoing pelvic radiotherapy, an inulin/FOS mixture showed a trend towards reducing the number of days with watery stools [97]. In moderate-to-severe acute pancreatitis, lactulose improves intestinal dysfunction and reduces systemic inflammation [11].
- Inflammatory Bowel Disease (IBD): Evidence points to a disease-activity-dependent effect. In active UC, prebiotics such as GOS and kestose have improved clinical activity scores and remission rates [81,100], with higher doses of ITF showing better clinical responses correlated with increased fecal butyrate production [96]. Interestingly, in Crohn’s disease, a prebiotic-induced increase in bifidobacteria was more pronounced in healthy siblings than in patients, suggesting that an inflamed environment may limit efficacy [95].
- Metabolic Disorders with GI Components: Prebiotics can address underlying dysbiosis. In children with overweight/obesity, oligofructose-enriched inulin is linked to reduced body fat and improved inflammatory markers [91]. In adults with metabolic syndrome, inulin-based interventions have led to beneficial microbial shifts and improved metabolic parameters [103].

### 6.4. Synthesis and Subgroup Analysis: Key Drivers of Prebiotic Efficacy

The analysis of the 22 RCTs revealed that while prebiotic interventions consistently modulate the gut microbiota (notably increasing *Bifidobacterium* abundance), the translation into clinical benefits was influenced by several intervention-specific variables. A systematic evaluation of these variables will help clarify which factors drive more consistent outcomes.

#### 6.4.1. Prebiotic Type and Structure

i. Inulin-Type Fructans (ITF: inulin, oligofructose, Synergy1): These were the most frequently studied prebiotics (11/22 studies). They demonstrated robust bifidogenic effects in the population. Clinical benefits were most pronounced for improving stool frequency and consistency in individuals with constipation [78,90,94] and for modulating metabolic parameters in overweight/obese children and adults with metabolic disorders [91,103]. The dose–response relationship suggested that higher doses (e.g., 15 g/d vs. 7.5 g/d) were associated with better clinical responses in patients with active UC [97].
ii. Galacto-oligosaccharides (GOS): GOS interventions (5/22 studies) also showed a strong and often dose-dependent bifidogenic effect [97,98,105]. Their clinical efficacy has been particularly noted in improving stool frequency in constipated adults 81 and modulating immune parameters in the elderly [105]. Interestingly, GOS showed promise in UC; however, its effects appeared to be dependent on baseline disease activity [81].
iii. Fructo-oligosaccharides (FOS): FOS (3/22 studies) effectively increased *Bifidobacterium* and improved constipation in infants [94]. In combination with other treatments (e.g., inulin), it showed trends in managing radiotherapy-induced enteritis [97].
iv. Other Prebiotics (lactulose, XOS, β2-1 fructan): Lactulose demonstrated significant benefits in acute clinical settings, improving intestinal dysfunction in acute pancreatitis [109] and aiding microbiome recovery after post-antibiotic treatment [108]. Specific compounds, such as β2-1 fructan, have been shown to exert direct immunomodulatory effects in healthy adults [104].

Taken together, the clinical evidence reveals distinct, although sometimes overlapping, efficacy profiles for the two most studied prebiotic subtypes, GOS and ITF. While both consistently exert a bifidogenic effect, GOS has demonstrated pronounced efficacy in improving stool frequency in adults with functional constipation [98] and modulating immune parameters, such as enhancing natural killer (NK) cell activity in the elderly [105]. Its benefits in UC also appear to be more contingent on baseline disease activity [81]. In contrast, ITF supplementation has shown a broader range of significant outcomes, including robust effects on stool frequency/consistency in constipation [78,90,94], modulation of metabolic parameters in overweight and metabolic syndrome populations [91,103], and a clearer dose–response relationship in active UC, where higher doses correlate with clinical improvement and increased fecal butyrate [96]. This distinction underscores that the choice between these major prebiotic types can be guided by the primary clinical targets.

#### 6.4.2. Dosage

A clear dose-dependent relationship was observed for certain outcomes. For constipation, 11 g/d GOS was effective in increasing stool frequency, whereas 5.5 g/d showed lesser effects [98]. In UC, a dose of 15 g/d ITF was superior to 7.5 g/d in inducing a clinical response [96]. For general bifidogenic effects, doses ranging from 5 to 16 g/day were commonly effective; however, optimal doses for specific clinical endpoints (e.g., metabolic improvement and immunomodulation) remain to be precisely defined.

#### 6.4.3. Duration of the Intervention

i. Short-term (≤4 weeks): Sufficient to induce significant microbial shifts (increased *Bifidobacterium* and SCFA production) and improve constipation symptoms [78,94,98].
ii. Medium-term (8–16 weeks): Required to observe metabolic improvements (e.g., reduced body fat and improved glycemic trends) [91,92] and more substantial clinical responses in chronic conditions such as UC [96,100].
iii. Long-term (>16 weeks): The data were sparse. One 24-week study in children demonstrated sustained bifidogenic effects and reduced infectious episodes [93], highlighting the potential for long-term preventive benefits of prebiotics.

#### 6.4.4. Population Characteristics

i. Baseline Health Status: The most consistent and pronounced clinical benefits (e.g., normalized stool patterns, reduced inflammation) were observed in healthy or mildly compromised individuals (e.g., simple constipation, healthy elderly individuals) and in specific dysbiotic states (antibiotic-associated, pediatric obesity). In contrast, responses in active IBD are more variable and appear to be contingent on baseline inflammation levels [81,95].
ii. Age: Children and the elderly showed strong bifidogenic responses. Clinically, children benefited in terms of infection prevention and constipation relief, whereas the elderly showed improved immune markers.

#### 6.4.5. Conclusions of Subgroup Analysis

The most consistent clinical benefits (improved bowel habits and bifidogenic effects) are driven by adequate doses (≥5–8 g/d) of well-established prebiotics (ITF and GOS) administered for at least 3–4 weeks in populations without severe baseline inflammation. To manage metabolic parameters or active mild-to-moderate UC, higher doses (≥12–15 g/d) and longer durations (≥8 weeks) of specific prebiotics (ITF, GOS) may be necessary. The significant role of the host baseline microbiota and disease activity as effect modifiers is evident, particularly in complex conditions such as IBD, underscoring the need for personalized approaches.

### 6.5. Limitations, Heterogeneity, and Implications for Evidence Interpretation

Despite the compelling narrative of efficacy emerging from this analysis, the interpretation and generalizability of prebiotic benefits must be critically tempered by the significant methodological limitations and heterogeneity of the existing literature. These constraints directly affect how key findings should be weighted and the strength of the conclusions that can be drawn from them.

The small sample sizes (typically n < 50) that characterize most trials not only increase the risk of Type II errors but also mean that the observed effect sizes, such as the degree of improvement in stool frequency or reduction in inflammatory markers, are estimated with low precision. Consequently, while the direction of the effect is often positive, the magnitude of the benefit for any specific outcome remains uncertain for broader populations. This limitation severely restricts the validity of subgroup analyses and the extrapolation of findings across different ages, ethnicities, and comorbidities.

The lack of rigorous blinding and placebo control in several pivotal studies, particularly those involving active disease states (e.g., IBD), introduces a high risk of performance and detection bias. In conditions with subjective endpoints (e.g., stool consistency and abdominal pain) or a known placebo response, such as irritable bowel syndrome and mild-to-moderate UC, this design flaw makes it difficult to attribute clinical improvements solely to prebiotic intervention. Therefore, the promising results seen in some open-label studies [81,96,103] must be considered preliminary and require confirmation in more stringent trials.

Furthermore, the short-term duration of the interventions (typically 3–12 weeks) has critical implications for interpreting their clinical relevance. While sufficient to demonstrate initial microbial shifts and acute symptomatic relief, these timeframes are inadequate to assess whether prebiotic-induced changes are sustainable, whether tolerance develops, or whether they translate into meaningful long-term health outcomes. For chronic conditions such as metabolic syndrome or IBD, short-term studies cannot determine whether prebiotics alter disease progression, prevent complications, or reduce relapse rates. The field lacks data on the long-term ecological stability of prebiotic-modulated microbiota and the corresponding durability of the clinical effects.

Closely related to the issue of short duration is the paucity of data on the long-term safety of sustained prebiotic use, specifically regarding the potential for unintended microbiota imbalance. While prolonged selective fermentation is intended to promote beneficial taxa, there is a theoretical risk of excessively reducing microbial diversity or promoting the overgrowth of other saccharolytic bacteria with less desirable metabolic profiles [88]. The 12-month RCT by Neumer et al. [110] in healthy infants demonstrated that oligofructose-enriched inulin was safe and sustained a bifidogenic effect without any reported adverse outcomes. However, this evidence is specific to early life development and a single prebiotic. Data in adult populations, particularly those with pre-existing dysbiosis (e.g., IBD, IBS), are virtually absent. The clinical implications are significant: without long-term safety data (>6–12 months), recommendations for continuous, lifelong prebiotic supplementation in chronic disease management remain premature, and optimal regimens (continuous versus cyclical) remain undefined.

Notably, the heterogeneity in interventions, including prebiotic type, dosage, and purity, precludes definitive conclusions about the superiority of any single compound for a given indication. This variability, while reflecting an exploratory phase of research, means that our synthesized findings represent a composite signal across various agents. The clinical community cannot yet be guided by standardized protocols, as the optimal “dose–response” and “structure–function” relationships for most endpoints remain poorly defined.

Finally, the reductionist approach to microbiome analysis and the frequent disconnect between taxonomic and functional data limit the mechanistic depth of these conclusions. The reliance on 16S rRNA gene sequencing of fecal samples provides a community census but offers limited insight into the functional capacity or activity of the microbiota, particularly in the mucosal niche of the gut. The failure to consistently link increases in putatively beneficial taxa (e.g., *Faecalibacterium*) with corresponding increases in their expected metabolites (e.g., butyrate) [95,105] highlights a significant knowledge gap in this area. This “taxonomy–function gap” complicates the interpretation of microbial data, as an increase in a specific bacterial group does not necessarily confirm the anticipated functional or health benefits. Future studies integrating metagenomics, transcriptomics, and metabolomics are essential to move beyond these correlations and establish causal pathways.

In conclusion, the current clinical evidence base, while promising and biologically plausible, is primarily composed of proof-of-concept and pilot studies with small sample sizes. The limitations of scale, design, duration, and measurement directly impact the strength of the evidence, confound cross-study comparisons, and restrict generalizability. These factors collectively underscore the fact that prebiotic research remains in the translational phase. Although the observed biological effects are consistent with bifidogenesis, they have not yet been uniformly matched by robust, reproducible, and clinically definitive outcomes across diverse populations. Therefore, the current findings should be interpreted as generating strong hypotheses for future research rather than providing definitive guidelines for clinical practice.

## 7. Future Directions and Considerations for Prebiotic Research

The promising yet preliminary evidence and methodological constraints summarized in Section 6.5 necessitate a decisive shift in research paradigms. To advance from suggestive findings to definitive personalized applications, a concerted agenda is required that directly addresses the core limitations of current studies while exploring novel mechanistic and translational approaches.

The paramount priority is to conduct large-scale, long-term randomized controlled trials (RCTs) with standardized protocols. Future studies must move beyond proof-of-concept pilots to definitively establish the optimal dosage, treatment duration, and specific prebiotic type (e.g., defined chain lengths of inulin or GOS) for specific health endpoints and patient populations to achieve this goal. This directly addresses the critical shortcomings of small sample sizes, short durations, and heterogeneous designs identified in previous studies. This effort must be coupled with a deeper mechanistic inquiry to understand how distinct prebiotic structures selectively influence microbial metabolism and host signaling pathways, which is essential for developing targeted interventions rather than generic ones.

To enable true personalization, a fundamental line of inquiry must focus on host-specific factors that determine response variability. Age is a key determinant of gut ecosystem structure and stability. Consequently, comparative research is urgently needed to elucidate the differential responses to prebiotic supplementation among children, adults, and older adults, whose immunological and physiological milieu differ markedly [59,77]. This demographic mapping should be integrated with investigations into how an individual’s baseline microbiota composition predicts clinical benefits, paving the way for biomarker-driven nutritional strategies.

Parallel to refining interventions for known benefits, the frontier of prebiotic research is expanding into novel physiological domains, most notably the gut–brain axis. Emerging evidence suggests that prebiotics may influence mental well-being and cognitive function via microbial modulation [7,49,72,111]. To validate this promising avenue, rigorously controlled trials are required to dissect the specific microbial and neurochemical pathways involved and determine whether prebiotics offer viable adjunctive strategies for managing mood and cognitive disorders.

The efficacy of any dietary intervention cannot be evaluated in a vacuum; it is inherently moderated by an individual’s overall dietary pattern. A crucial yet underexplored area is the interaction between prebiotic supplementation and background diets, such as plant-based and omnivorous diets. Therefore, future studies should examine whether the prebiotic effects are potentiated, attenuated, or qualitatively altered in different dietary contexts. This line of inquiry will provide essential guidance for tailoring recommendations to individual dietary preferences and cultural practices in the future.

### 7.1. Rational Design of Synbiotics for Targeted Microbiome Modulation

A logical progression in microbiome-targeted nutrition is the strategic development of synbiotics, which are synergistic combinations of specific probiotics and prebiotics designed to confer enhanced health benefits compared to either component administered alone [112]. Moving beyond arbitrary combinations toward a rationally designed framework represents a key frontier for achieving robust and predictable modulation of the gut ecosystem.

The efficacy of synbiotics depends on the complementary metabolic pathways. A prime example is the pairing of *Bifidobacterium* strains with either FOS or GOS. Bifidobacteria possess specialized enzymes, such as β-fructosidases and β-galactosidases, which efficiently hydrolyze these prebiotics, providing a selective growth advantage [113]. This synergy translates into clinical benefits for patients. For instance, a triple-blind trial in elderly patients with type 2 diabetes and high cardiovascular risk demonstrated that a multi-species synbiotic led to significant improvements in glycemic control (HbA1c), lipid profiles, and inflammatory markers [114]. Similarly, the pairing of β-GOS with *L. lactis* subsp. *lactis* enhanced the in vitro antimicrobial effects, as both prebiotics fueled the probiotic and upregulated its bacteriocin production [59].

In addition to nutritional support, prebiotics in synbiotic formulations can modulate probiotic fitness. For example, inulin enhances the adhesion of certain *Lactobacillus* strains to intestinal mucus in vitro, potentially improving their persistence and activity in the gut [115]. This mechanistic synergy extends to clinical outcomes; a meta-analysis concluded that synbiotics were more effective than probiotics alone in reducing the duration of acute diarrhea in children, partly due to improved probiotic survival during gastrointestinal transit [116].

Future synbiotic research should prioritize rational design. This involves (1) selecting probiotic strains with proven genomic capacity to utilize the companion prebiotic (in silico and in vitro screening); (2) validating that the prebiotic selectively enhances the probiotic’s survival, colonization, or production of beneficial metabolites (e.g., SCFAs); and (3) conducting head-to-head clinical trials against stand-alone components to demonstrate superior efficacy for specific health endpoints [89,112]. Investigating synbiotics in specific dysbiosis-related conditions, such as UC, where both microbial supplementation and metabolic fuel are critical, is particularly promising [109].

In conclusion, synbiotics represent a paradigm shift from broad-spectrum to precision microbiome interventions. Harnessing diet–microbe interactions at the mechanistic level offers a powerful strategy for achieving more profound and stable shifts in the gut microbiota, translating into enhanced clinical benefits. The rational development of these compounds should be prioritized in future personalized nutrition strategies.

### 7.2. Translational and Ethical Considerations

All these scientific endeavors must be underpinned by proactive engagement with translational and ethical considerations to ensure their success. As the potential of microbiome-based nutrition gains public traction, the commercialization and marketing of prebiotic and synbiotic products must be guided by rigorous scientific evidence. This involves developing clear regulatory frameworks for health claims and fostering transparent communication to distinguish evidence-based applications from hype, thereby protecting public trust and ensuring equitable access to genuine health innovations.

Finally, this research paradigm must address the intrinsic complexity of prebiotic sources. While diverse forms of these compounds are available, ranging from natural foods (e.g., garlic and onions) to refined supplements [26], their comparative efficacy, required doses, and impacts on the gut microbiota have not been fully characterized. A critical future direction is to conduct head-to-head comparisons that elucidate whether the whole-food matrix confers advantages over purified extracts and defines source-specific applications within the personalized nutrition framework.

## 8. Conclusions

This review consolidates evidence that prebiotics, through their specific structural properties, selectively modulate the gut microbiota and enhance SCFA production, translating into clinical benefits for the gastrointestinal and metabolic health of the host. However, significant inter-individual variability and methodological heterogeneity in intervention design (type, dose, duration) limit robust consensus and clinical generalizability of these findings.

To translate this promising evidence into actionable guidelines, future research must prioritize the following specific evidence-derived goals:

i. Defining Standardized and Precision Protocols: Conduct large-scale, long-term RCTs that directly compare specific prebiotic structures (e.g., different DP inulin) at defined doses to establish causal dose–response relationships and optimal regimens for target conditions (e.g., ≥15 g/d ITF for active UC and ≥8 weeks for metabolic improvement).
ii. Deciphering the Determinants of Response: Moving beyond taxonomy by integrating longitudinal multi-omics data (metagenomics and metabolomics) with deep host phenotyping to identify predictive biomarkers of response. This will elucidate why “responders” and “non-responders” exist and enable biomarker-driven treatment in personalized medicine.
iii. Validating Novel Mechanistic Pathways: Rigorously test emerging applications, such as gut–brain axis modulation for mood disorders, through mechanistic human trials that measure specific neuroactive metabolites (e.g., peripheral BDNF and GABA) alongside microbial changes.
iv. Rational synbiotic formulations: Advancing from generic combinations to mechanistically synbiotic pairs, where a prebiotic is selectively utilized by a co-administered probiotic strain, demonstrating superior efficacy to either component alone in head-to-head trials for specific dysbiotic states.

Addressing these priorities will bridge the gap between mechanistic promise and definitive personalized prebiotic interventions in clinical practice.

## Figures and Tables

**Table 1:** Characteristics and primary outcomes of clinical studies on prebiotic interventions targeting gastrointestinal and metabolic health.

Study Design	Sample Details	Experimental Groups	Treatment Schedule	Microbiome Data	Clinical Outcomes	Limitations	Reference
**Pediatric/Neonatal**							
RCT, DB, PC	34 children with celiac disease (CeD) on GFD (4–18 y, 62% F)	Synergy 1 (10 g/d oligofructose-enriched inulin, *n* = 18) vs. Placebo (maltodextrin, *n* = 16)	3 months, daily oral	↑ *Bifidobacterium*; stable *C. leptum*; ↓ *Lactobacillus*; ↑ total SCFAs (31%), acetate, butyrate in Synergy 1 group	No major AEs; improved stool consistency (95% vs. 69% normal stools in placebo)	Small n; wide age range; short duration; no clinical symptom scoring	[90]
RCT, DB, PC, parallel	42 OW/OB children (7–12 y, BMI ≥ 85th %ile)	Prebiotic: OI (8 g/d, *n* = 22) vs. Placebo: maltodextrin (3.3 g/d, 20)	16 wks (dose ↑ after 2-wk adapt.), before dinner	↑ *Bifidobacterium* spp., ↓ *Bacteroides vulgatus*; ↔ α-diversity; β-diversity changed in OI only	↓ body weight z-score, ↓ total body fat %, ↓ trunk fat %, ↓ IL-6, ↓ triglycerides; weight gain normalized	Homogeneous sample (white, mid/high SES); stool collection not time-controlled; modest n	[91]
RCT, DB, PC, parallel pilot	43 children with T1D (8–17 y, duration > 1 y, HbA1c < 10%)	Prebiotic: OI (8 g/d, *n* = 17) vs. Placebo: maltodextrin (3.3 g/d, *n* = 21)	12-wk intervention + 3-mo washout; half dose first 2 wk	↑ *Bifidobacterium*; ↓ α-diversity (Shannon); β-diversity altered; placebo ↑ *Streptococcus*, *Roseburia inulinivorans*	↔ HbA1c; ↑ C-peptide in prebiotic vs. placebo (*p* = 0.029); ↓ intestinal permeability trend (*p* = 0.076)	Small n; short intervention; no HbA1c improvement; mostly white cohort; compliance < 80% in some	[92]
RCT, DB, PC, multi-center	258 healthy children (3–6 y) attending kindergarten	Prebiotic: ITF (6 g/d, *n* = 104) vs. Placebo: maltodextrin (*n* = 105)	24 weeks, daily	↑ *Bifidobacterium* & *Lactobacillus*; ↑ bifidobacteria sustained during/after antibiotics; 3 enterotypes; prebiotic attenuated antibiotic-induced ↓ in bifidobacteria	Reduced febrile episodes requiring medical attention; ↓ incidence of sinusitis; ↔ total bacteria, *C. difficile*, *C. perfringens*, Enterobacteriaceae	Different antibiotics prescribed (not analyzed separately); results partly from subgroup with antibiotics; enterotype stability not assessed long-term	[93]
RCT, DB, parallel	36 infants (6–24 mos) with constipation	FOS (weight-based, 6–12 g/d, *n* = 18) vs. Placebo (maltodextrin, *n* = 18)	4 weeks	↑ *Bifidobacterium* spp. in FOS group (*p* = 0.006) vs. placebo; *Lactobacillus* spp. NS	Softer stools (*p* = 0.035), ↓ straining/difficulty (*p* = 0.041), ↓ transit time (*p* = 0.035); therapeutic success ↑ (83.3% vs. 55.6%, NS)	Small n; high placebo response; dietary changes during study may confound	[94]
**Gastrointestinal Conditions**							
Interventional pilot	19 CD patients in remission (CDAI < 150) & 12 unaffected siblings	Patients vs. Siblings (all received oligofructose/inulin)	15 g/d × 3 weeks	Siblings: ↑ *B. longum*, *B. adolescentis*, *Roseburia* spp. Patients: ↑ *B. longum* only	↔ Calprotectin; ↓ intestinal permeability in patients (esp. ileal CD); ↓ blood CD3^+^ T cells in siblings	No placebo; small n; no unrelated healthy controls; most patients on stable meds	[95]
Dose-finding, open-label	25 adults with mild/moderate active UC	7.5 g/d vs. 15 g/d Orafti^®^Synergy1 (oligofructose-enriched inulin)	9 weeks	15 g/d: ↑ Bifidobacteriaceae & Lachnospiraceae in feces; ↓ Bacteroidaceae and Porphyromonadaceae in mucosa	15 g/d: ↑ clinical response (77% vs. 33%, *p* = 0.04) and remission; ↓ Mayo score and calprotectin; ↑ fecal butyrate (corr. with ↓ Mayo, r = −0.50)	No placebo; small n; open-label; most patients on 5-ASA	[96]
RCT, DB, PC, crossover	42 healthy adults with mild constipation (per protocol)	12 g/d chicory inulin vs. maltodextrin placebo	4 weeks per intervention, 2-wk run-in, 4-wk washout, crossover	↑ *Bifidobacterium* and *Anaerostipes*; ↓ *Bilophila*; ↔ α-diversity; ↓ richness; ↔ enterotype; 0.8% treatment effect on global microbiota	↑ stool frequency; softer stools; ↓ *Bilophila* linked to improved PAC-QoL (physical discomfort & treatment satisfaction)	Metabolomics limited to volatile compounds; no SCFA/lactate measurement; healthy mildly constipated only; GC-MS may miss polar metabolites	[78]
RCT, DB, PC, parallel	38 women with gynecological cancer (post-op + pelvic RT)	Prebiotic: 6 g bid mixture (50% inulin + 50% FOS, *n* = 19) vs. Placebo: 6 g bid maltodextrin (*n* = 19)	~9 weeks (1 wk pre-RT → throughout RT → 3 wk post-RT)	Not reported here (earlier study: ↑ recovery of *Lactobacillus* and *Bifidobacterium* post-RT)	↓ days with watery stools (Bristol 7) in prebiotic group (*p* = 0.08); ↔ number of BMs; ↔ diarrhea grade; ↔ QoL scores (EORTC-QLQ-C30)	Small n; subjective stool diary; no microbiome data in this report; high dropout rate (10/48)	[97]
Prospective single-center RCT	73 adults with MSAP + intestinal dysfunction (SGD > 5)	Lactulose (*n* = 36) vs. Rhubarb (*n* = 37)	Lactulose: 50 mL bid for 1 wk, then 10 mL bid after GI recovery; Rhubarb: 50 g bid for 1 wk	↑ *Bifidobacterium* (lactulose); ↑ *Escherichia-Shigella* (rhubarb); ↑ SCFAs (both, stronger with lactulose)	GI function improved in both (SGD ↓ to 0); no diff in infectious complications, organ failure, LOS; ↓ TNF-α, IL-6 in lactulose group	Small n; 16S rRNA only; no placebo; antibiotics confound microbiota results	[11]
RCT, DB, PC, parallel	132 adults with constipation (Rome IV), 94% F	11 g GOS (*n* = 44), 5.5 g GOS (*n* = 45), Placebo (maltodextrin, *n* = 43)	Once daily for 3 weeks	↑ *Bifidobacterium* (dose-dependent); ↑ *Anaerostipes hadrus* (11 g GOS)	↑ stool frequency in 11 g GOS vs. placebo in subjects with ≤3 BM/week (*p* = 0.027) and in adults ≥ 35 yrs (*p* = 0.010); ↔ stool consistency or SCFA	High placebo response; many subjects had >3 BM/week at baseline; short intervention; no transit time measured	[98]
Two RCTs, DB, PC, crossover	50 healthy adults with low dietary fiber intake, BMI 18.5–29.9	Trial 1: Moderate-dose bar (7 g ITF/d, *n* = 25) vs. Control 1; Trial 2: Low-dose bar (3 g ITF/d, *n* = 25) vs. Control 2	One snack bar daily for 4 weeks, with 4-week washout	↑ *Bifidobacterium* (significant with 7 g/d, trend with 3 g/d); ↑ Actinobacteria; ↓ *Firmicutes*; predicted functional changes with 7 g/d only	↑ total fiber intake; ↔ weight, GI symptoms, stool consistency, or QoL; minor within-group ↑ in bloating (all groups)	Fecal SCFA not different; mainly Caucasian, higher SES; no blood SCFA; symptoms possibly placebo-influenced	[99]
RCT, DB, PC pilot	40 patients with mild-moderate active UC	G1: 1-kestose (*n* = 20) vs. G2: placebo (maltose, *n* = 20)	Oral for 8 weeks + standard treatment	↓ α-diversity; ↓ *Ruminococcus gnavus* group; SCFAs ↔	Lichtiger CAI ↓ (3.8 vs. 5.6, *p* = 0.026); remission 55% vs. 20% (*p* = 0.048); endoscopic score ↔	Small n; endoscopic improvement not significant; short duration (8 wk); SCFAs unchanged	[100]
Open-label, pre–post	17 adults with mildly active UC (SCCAI > 0, calprotectin > 150 µg/g or endoscopic evidence)	All received GOS (2.8 g/d)	6 weeks, once daily (dissolved in 300 mL water)	↑ *Bifidobacterium* & ↑ Christensenellaceae only in baseline SCCAI ≤2 subgroup (*p* < 0.05); ↓ *Dialister* in SCCAI >2 subgroup; No Δ in α-diversity or SCFAs	↑ normal stool proportion (BSFS) (49% → 70%, *p* = 0.024); ↓ loose stool incidence (GSRS) (3.2 → 1.6 days, *p* = 0.012) & severity (0.7 → 0.5, *p* = 0.048); ↓ urgency (SCCAI) (1.0 → 0.5, *p* = 0.011); No Δ in SCCAI total score or fecal calprotectin	No control group; small n; disease activity heterogeneity; short duration; open-label design	[81]
**Metabolic Disorders**							
RCT, DB, PC, crossover	25 patients with T2D (15 M, 10 F), on metformin (68%)	16 g/d ITF (50:50 oligofructose:inulin) vs. 16 g/d maltodextrin placebo	6 weeks per intervention, 4-week washout, crossover	↑ *Bifidobacterium adolescentis* OTUs; ↔ microbial diversity; ↑ total SCFA, acetate, propionate; ↔ butyrate	Not reported (study focused on microbiota and SCFA)	Fecal SCFA not fully representative of colonic production; 6 weeks may be too short for diversity changes; high baseline fibre intake	[101]
RCT, DB, PC, crossover	12 HD patients (6 M, 6 F), 50% Black American, 33% with diabetes	Inulin (F: 10 g/d, M: 15 g/d) vs. maltodextrin (F: 6 g/d, M: 9 g/d)	4 weeks per intervention, 4-week washout, crossover	↑ *Akkermansia* (interaction *p* = 0.045); ↔ *Bifidobacterium*; ↑ Bacteroidetes/*Bacteroides* (time effect); ↔ α- and β-diversity; ↑ fecal acetate and propionate (time effect)	↔ plasma p-cresyl sulfate & indoxyl sulfate; ↔ fecal p-cresol & indoles	Small n; high BMI; placebo (maltodextrin) unexpectedly increased SCFA; high inter-individual variability; no breath test for adherence	[102]
Open-label pilot RCT (single-blinded)	60 adults with MetS (26M, 34F)	3 groups: Inulin, Inulin + TCM, Inulin + Metformin (20/group)	6 months	Inulin alone: ↑ *Bacteroides*; Inulin + TCM: ↑ *Romboutsia*; Inulin + Met: ↑ *Streptococcus* and ↑ *Holdemanella*; ↔ α-diversity	Primary focus on microbiome modulation	Open-label design. No true placebo control.	[103]
**General Health/Immunity**							
RCT, DB, PC, crossover	30 healthy adults (17F, 13M), age ~28 y, BMI ~24 kg/m^2^	β2-1 fructan (Synergy1, 15 g/d) vs. Placebo (maltodextrin, 15 g/d)	2 phases of 28 d each, 14-d washout (3 × 5 g/d with meals)	↑ *Bifidobacterium* (~3-fold); ↑ total SCFA, propionate, butyrate; ↓ acetate & BCFA	Immunomodulation: ↑ serum IL-4, GM-CSF, TLR2+ mDCs; ↓ IL-10. Symptoms: ↑ GI discomfort (bloating, gas). No Δ: lipids, CRP, Ig, well-being.	Small n; short duration; no clear health benefit in healthy adults; increased GI symptoms; immune changes of unclear clinical significance.	[104]
RCT, DB, PC, parallel, cross-over	40 elderly (25F, 15M), age 65–80 y	B-GOS (5.5 g/d) vs. maltodextrin placebo	10-week intervention, 4-week washout	↑ *Bifidobacterium* spp., ↑ *Bacteroides–Prevotella*, ↔ other taxa	↑ NK cell activity; ↑ IL-10, IL-8, CRP; ↓ IL-1β; ↔ bowel habits	Single GOS product; metabolic changes partly diet-confounded; CRP rise (though low-level)	[105]
RCT, DB, PC, parallel	26 elderly (≥55 y), healthy	Long-chain inulin (8 g/d) vs. glucose placebo (5 g/d)	9-week intervention (8-week supplement + 1-week post)	↑ α-diversity; ↑ *Bifidobacterium* spp. (*B. adolescentis*, *B. angulatum*, *B. ruminantium*); ↑ *Alistipes shahii*, *Anaerostipes hadrus*, *Parabacteroides distasonis*; ↔ SCFA in feces (↓ isobutyrate trend)	↔ anti-HB antibody titers; ↔ T-cell subsets; ↔ vaccine responder rate	Small n; immune outcomes unchanged; fecal SCFA may not reflect proximal production	[106]
RCT, DB, PC, dose–response	80 healthy adults (18–55 y), Indian, lower SES	Placebo (maltodextrin 10 g/d), FOS 2.5 g/d, FOS 5 g/d, FOS 10 g/d	90-day dosage phase (daily), 9 timepoints over 210 days	↑ *Bifidobacterium* and *Lactobacillus* (both FOS and placebo); ↑ diversity (FOS), ↓ after withdrawal; ↑ butyrate producers (*Faecalibacterium*, *Ruminococcus*, *Oscillospira*)	Random glucose ↔, calcium ↑ slightly, triglycerides ↔; effects reversible after cessation	Diet not strictly controlled; no functional metagenomics	[107]
RCT, DB	87 children (3–14 y) with respiratory infections	G1: azithromycin + lactulose (*n* = 44) vs. G2: azithromycin only (*n* = 43)	3-day treatment, follow-up at 18 ± 2 d and 60 ± 2 d	G1: ↑ *Lactobacillus*, *Enterococcus*, *Anaerostipes*, *Blautia*, *Roseburia*; ↓ *Prevotella*; G2: ↑ opportunistic pathogens (*Streptococcus*, *Veillonella*) at 60 d; ↓ *Enterobacter*	Microbiome restoration faster in G1; G2 showed prolonged dysbiosis & opportunistic pathogen rise	No clinical symptom monitoring; short follow-up (60 d); age range broad; no placebo group	[108]

AE: Adverse Event, BCFA: Branched-Chain Fatty Acid, BM: Bowel Movements, BMI: Body Mass Index, BSFS: Bristol Stool Form Scale, CAI: Clinical Activity Index, CD: Crohn’s Disease, CeD: Celiac Disease, DB: Double-Blind, FOS: Fructo-oligosaccharides, GFD: Gluten-Free Diet, GI: Gastrointestinal, GOS: Galacto-oligosaccharides, GSRS: Gastrointestinal Symptom Rating Scale, HbA1c: Glycated Hemoglobin, HD: Hemodialysis, Ig: Immunoglobulin, IL: Interleukin (e.g., IL-4, IL-10), ITF: Inulin-Type Fructans, LOS: Length of Hospital Stay, mDC: Myeloid Dendritic Cell, MetS: Metabolic Syndrome, MSAP: Moderately Severe Acute Pancreatitis, n: Sample Size, NK: Natural Killer, NS: Not Significant, OB: Obese, OI: Oligofructose-Enriched Inulin, OW: Overweight, PAC-QoL: Patient Assessment of Constipation Quality of Life, PC: Placebo-Controlled, QoL: Quality of Life, RCT: Randomized Controlled Trial, RT: Radiotherapy, SCFA: Short-Chain Fatty Acid, SCCAI: Simple Clinical Colitis Activity Index, SES: Socioeconomic Status, SGD: Score of Gut Dysfunction, T1D: Type 1 Diabetes, T2D: Type 2 Diabetes, TCM: Traditional Chinese Medicine, TLR: Toll-like Receptor, TNF-α: Tumor Necrosis Factor-Alpha, UC: Ulcerative Colitis, Δ: Change, ↔: No Change, ↑: Increased, ↓: Decreased.

## Data Availability

No new data was created.

## Abbreviations

The following abbreviations are used in this manuscript:

AE	Adverse Event
AMX	Amoxicillin
AP	Acute Pancreatitis
BBB	Blood–Brain Barrier
BCAA	Branched-Chain Amino Acids
BCFA	Branched-Chain Fatty Acids
BDNF	Brain-Derived Neurotrophic Factor
BMI	Body Mass Index
BM	Bowel Movements
CD	Crohn’s Disease
CeD	Celiac Disease
CLA	Conjugated Linoleic Acid
CRP	C-Reactive Protein
DP	Degree of Polymerization
FOS	Fructo-oligosaccharides
GABA	Gamma-Aminobutyric Acid
GFD	Gluten-Free Diet
GI	Gastrointestinal
GOS	Galacto-Oligosaccharides
HbA1c	Glycated Hemoglobin
HFD	High-Fat Diet
HMOs	Human Milk Oligosaccharides
HOMA	Homeostatic Model Assessment
HPA axis	Hypothalamic–Pituitary–Adrenal Axis
IBD	Inflammatory Bowel Disease
Ig	Immunoglobulin
IL	Interleukin (e.g., IL-1, IL-6, IL-10)
ISAPP	International Scientific Association for Probiotics and Prebiotics
ITF	Inulin-Type Fructans
LOS	Length of Hospital Stay
mDC	Myeloid Dendritic Cell
MetS	Metabolic Syndrome
MSAP	Moderately Severe Acute Pancreatitis
NK cell	Natural Killer cell
NS	Not Significant
OB	Obese
OW	Overweight
PAC-QoL	Patient Assessment of Constipation Quality of Life
QoL	Quality of Life
RCT	Randomized Controlled Trial
RS	Resistant Starch
RT	Radiotherapy
SCFA(s)	Short-Chain Fatty Acid(s)
SCCAI	Simple Clinical Colitis Activity Index
SGD	Score of Gut Dysfunction
T1D	Type 1 Diabetes
T2D	Type 2 Diabetes
TNF-α	Tumor Necrosis Factor-Alpha
UC	Ulcerative Colitis
XOS	Xylo-Oligosaccharides
